# Dystropathology Increases Energy Expenditure and Protein Turnover in the Mdx Mouse Model of Duchenne Muscular Dystrophy

**DOI:** 10.1371/journal.pone.0089277

**Published:** 2014-02-19

**Authors:** Hannah G. Radley-Crabb, Juan C. Marini, Horacio A. Sosa, Liliana I. Castillo, Miranda D. Grounds, Marta L. Fiorotto

**Affiliations:** 1 CHIRI Biosciences Research Precinct, School of Biomedical Sciences, Curtin University, Perth, Australia; 2 School of Anatomy, Physiology and Human Biology, the University of Western Australia, Perth, Australia; 3 USDA/ARS Children’s Nutrition Research Center, Department of Pediatrics, Baylor College of Medicine, Houston, Texas, United States of America; 4 Pediatric Critical Care Medicine, Department of Pediatrics, Baylor College of Medicine, Houston, Texas, United States of America; Rutgers University -New Jersey Medical School, United States of America

## Abstract

The skeletal muscles in Duchenne muscular dystrophy and the mdx mouse model lack functional dystrophin and undergo repeated bouts of necrosis, regeneration, and growth. These processes have a high metabolic cost. However, the consequences for whole body energy and protein metabolism, and on the dietary requirements for these macronutrients at different stages of the disease, are not well-understood. This study used juvenile (4- to 5- wk-old) and adult (12- to 14-wk-old) male dystrophic C57BL/10ScSn-mdx/J and age-matched C57BL/10ScSn/J control male mice to measure total and resting energy expenditure, food intake, spontaneous activity, body composition, whole body protein turnover, and muscle protein synthesis rates. In juvenile mdx mice that have extensive muscle damage, energy expenditure, muscle protein synthesis, and whole body protein turnover rates were higher than in age-matched controls. Adaptations in food intake and decreased activity were insufficient to meet the increased energy and protein needs of juvenile mdx mice and resulted in stunted growth. In (non-growing) adult mdx mice with less severe dystropathology, energy expenditure, muscle protein synthesis, and whole body protein turnover rates were also higher than in age-matched controls. Food intake was sufficient to meet their protein and energy needs, but insufficient to result in fat deposition. These data show that dystropathology impacts the protein and energy needs of mdx mice and that tailored dietary interventions are necessary to redress this imbalance. If not met, the resultant imbalance blunts growth, and may limit the benefits of therapies designed to protect and repair dystrophic muscles.

## Introduction

Duchenne muscular dystrophy (DMD) is an X-linked lethal muscle disease that affects approximately 1 in 3500 male births [1], [2]. Mutations or deletions in the dystrophin gene compromise the integrity of the sarcolemma-associated dystrophin-glycoprotein complex and render dystrophin-deficient myofibers susceptible to damage upon contraction [3], [4]. Consequently, dystrophic skeletal muscles undergo repeated bouts of myofiber necrosis, regeneration and growth, processes with a high metabolic cost. The dystropathology is associated with inflammation, increased intracellular calcium, oxidative stress and metabolic abnormalities [5]–[10]. In DMD the heart is affected somewhat later than skeletal muscle, and results in cardiac complications that exacerbate the disease (reviewed in [1], [11]). The extent to which the heart is affected by changes in functional demand incurred by the altered metabolic demands is unclear. Overall, the cumulative costs of dystropathology on whole body protein and energy metabolism are not well-understood. Thus, the specific dietary protein and energy requirements of DMD boys remain poorly defined. In order to develop better guidelines for the nutritional management of DMD boys at different disease stages, a precise understanding of the metabolic consequences of the dystropathology and how these change with disease progression is urgently required.

Published data are inconsistent with regard to whether there is an increase or decrease in energy expenditure (EE) in DMD boys; values that span the gamut, from hyper- [12]–[14] to hypo-metabolic [15], [16] have been reported (reviewed in [17]–[19]). This discrepancy can be attributed to small sample sizes, non-standardised protocols for both data collection and normalization, and predominantly to the wide variation in disease severity among individual patients. Additionally, differences in age, ambulatory state, body composition, and ventilation contribute to variability. Defining the metabolic consequences of the dystropathology is further complicated by the side-effects of widely used corticosteroids [20].

These variables are indeed difficult to standardize in humans and, hence, there is a need for studies in animal models. This study analyses the mdx (C57BL/10ScSn-mdx/J) mouse model of DMD that has been used in many pre-clinical experiments [11], [21]–[23]. Despite differences in the long-term consequences and severity between mdx mice and DMD boys (that may be largely accounted for by the much longer duration of the growth phase, lifespan, and scale of humans [24], [25]), the mdx model offers some significant advantages. First, invasive measurements can be performed at different ages and disease stages. Moreover, the effects of the disease can be studied in the absence of corticosteroids or other therapies that modify the disease or induce their own metabolic effects. Additionally, the model provides the possibility of assessing the consequences of both extensive and relatively low-grade muscle degeneration and repair on whole body protein and energy metabolism.

**Figure 1:**
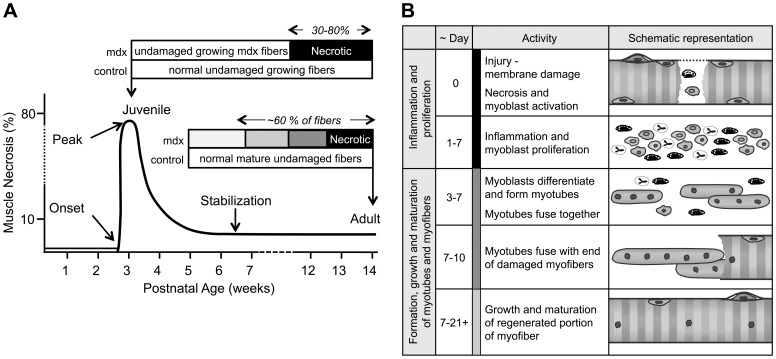
The progression of cellular events that occurs in dystrophic mdx limb muscles. (**A**) Time line showing the proportion of muscle fibers undergoing necrosis and regenerative events from birth to adulthood. There is little if any necrosis in the limb muscles of mdx mice prior to 3 wk of age [29], [73] when an abrupt onset of myofiber necrosis occurs [74]–[76]. By 4 wk of age approximately 30%–80% of the mdx muscles are characterized by inflammation, phagocytosis of necrotic tissue and early myogenesis; the rest of the myofibers remain essentially ‘intact’ [21], [73]. From 4 to 5 wk of age, myotubes form and damaged myofibers undergo regeneration [77]. In control mice myofibers are undamaged and grow by elongation and hypertrophy. After this initial bout of necrosis and as normal growth decelerates, the necrosis abates to a persistent low level (∼6%) from 8 to 12 wk of age [21], [26], [75]. However, even if only 3% of adult mdx myofibers undergo necrosis every day, ∼20% of the muscle tissue will be subjected to necrosis over one week. As the cycles of necrosis and subsequent active regeneration take about 3 wk, it can be calculated that ∼60% of adult mdx myofibers at any time are actively affected by necrosis and regeneration. (**B**) The time course of regenerative events after a single bout of myofiber necrosis. Within 1 d of damage, myofiber necrosis is evident histologically as fragmented sarcoplasm and the presence of inflammatory cells. Coincidently there is activation of myoblast proliferation followed by their differentiation and fusion into myotubes 3 to 7 d later [74], [78], [79]. The myotubes fuse with more myoblasts and each other over the next few days and after about 7 d, they fuse with the ends of the damaged myofibers [80]; coincidently, inflammatory cells decrease. Subsequently, myotubes and newly repaired segments of damaged myofibers hypertrophy and mature and may take a further 14 d [81]. Subsequently, the repaired fibers hypertrophy and undergo maturation attaining a stable adult size after approximately 3 weeks. Regenerated myofibers are identified by the presence of central myonuclei that persist for many months.

The severity of dystropathology varies throughout the lifespan of the mdx mouse and amongst mdx muscles. There is a classic sequence of cellular events in mdx muscles that has been extensively described in the literature (reviewed in [21], [23], [26]) beginning at 3 wks of age; this has also been confirmed for the mdx mice from our mouse colony [27]. An understanding of these events was fundamental to our study design and data interpretation. In Figure 1, generated from a synthesis of observations made in a number of laboratories (including our own), the two ages we selected to study (juvenile and adult) are identified. In the mdx diaphragm, the onset of myonecrosis is evident by ∼2 wk of age. Initially, it is less severe than in limb muscles, but it is sustained and chronically results in fibrosis and later resembles more closely the muscles of DMD boys [28]–[31]. The ages and muscles we have studied follow the recommendations of numerous international researchers and the TREAT-NMD Neuromuscular Network that attempt to standardize pre-clinical studies in the mdx mouse [21], [23].

Although the energy requirements of resting normal skeletal muscle are low relative to other organs in the body [32], [33], it needs to be considered that skeletal muscle constitutes about 40% or normal body mass. When there is a muscle dystropathology, protein turnover and ion transport, both high energy-consuming processes, are likely to be greater than normal. Thus, whether the enhanced activation of these processes is sufficient to increase total EE will depend on disease severity, the extent of necrosis/inflammation, and the proportion of regenerating and growing myofibers. Measurements of the effects of muscle dystropathology on EE in the mdx mouse have yielded ambiguous results. In older mice (6 to 12 mo), two studies found no differences between the EE of mdx and control mice [34], [35]. The significance of these studies is difficult to assess: differences in body composition were not accounted for; total versus resting metabolic rates, an essential first step for identifying the basis of differences in total EE, were not distinguished [34]; measurements were performed in multiply–housed mice and were too short to provide an accurate assessment of energy balance [35]; combined male and female mice were studied [34] or the gender of mice was not stated [35]. In young 4- to 6-wk-old mdx mice, where rates of muscle necrosis and regeneration are very high, a decrease in whole body metabolic rate was observed and attributed to reduced physical activity; however the energy balance data in this study do not explain the lower weights of the younger mdx mice [34].

Altered muscle protein turnover rates have been documented in various mdx muscles both *in vivo* and *ex vivo* [36]–[38]. High rates of protein synthesis have been demonstrated in gastrocnemius mdx muscles, but these data did not distinguish between the synthesis of muscle-specific proteins (indicative of regenerating myofibers) and other protein populations in muscles, such as the extracellular matrix proteins and those of non-muscle cells. Importantly, however, we do not know if these changes in muscle protein metabolism are sufficient to impact whole body protein turnover, and hence dietary protein requirements.

The current study aims to resolve the uncertainties generated by these earlier data, and to comprehensively define how dystropathology impacts protein and energy metabolism by measuring in individual mice the numerous factors that contribute to the variability in these parameters. This study tests the hypothesis that the process of dystrophic muscle necrosis and regeneration incurs significant energy costs that the organism attempts to meet by altering voluntary EE and food intake. We further hypothesize that when these adaptations do not counterbalance the increased nutrient needs incurred by the dystropathology, growth and maintenance of normal body composition become compromised. The extensive range of measurements performed encompass: food intake, EE, spontaneous cage activity, body composition, muscle protein synthesis rate - for both total (TP) and the myofibrillar protein (MP) fraction, and whole body protein turnover. Measurements were compared for mdx and normal mice, using juvenile 4- to 5-wk-old mdx mice during the early stages of muscle necrosis, regeneration and growth, and adult 12- to 14-wk-old mdx mice when myofibers exhibit a decreased, but ongoing, low chronic level of necrosis, regeneration and growth of repaired myofibers. Because the severity of dystropathology differs between limb, diaphragm, and heart muscles, all three were examined to broaden the assessment.

## Materials and Methods

### Animals

Adult male and female mdx (C57BL/10ScSn-mdx/J) mice and C57BL/10ScSn/J controls (Jackson Laboratory, Bar Harbor, ME) housed in polycarbonate cages with wood-chip bedding were mated. On postnatal day 2, litters were standardised to 6–7 pups per dam. At 21 d of age, male mice were individually housed in wire bottom cages with ad libitum access to food and water. They were then transferred to CLAMS cages (see below); measurements were made between 28 and 33 d of age in the “juvenile” group. Adult, 10-wk-old male mice were purchased or obtained from the in-house colony and left for a minimum of 2 wk to stabilize. They were individually housed in wire bottom cages with ad libitum access to food and water. All measurements were performed between 12 and 14 wk of age. An additional set of 14-wk-old control and mdx mice was used only for EE and activity measurements because a complete set of activity measurements was not obtained on the first set of mice. There was no difference in EE between the two replicates, and the data were pooled. Animal holding rooms were maintained at 23^o^C with a 12 h light/dark cycle (light from 06∶00 to 18∶00). Experiments were carried out according to the recommendations in the Guide for the Care and Use of Laboratory Animals of the National Institutes of Health and approved by the Baylor College of Medicine IACUC.

### Body Composition

Total fat-free (FFM) and fat masses were measured by dual-energy X-ray absorptiometry (DXA) using a PIXImus (General Electric). Correction factors derived specifically for our instrument were applied to fat and FFM estimates to adjust for the inherent calibration error (http://www.bcm.edu/cnrc/mmru/piximus). Body “length” was obtained from the sum of leg and spine lengths determined from the PIXImus-generated X-ray density image of each mouse and corrected for magnification by comparison to an object of known dimensions measured on the same plane as the mouse. For normalization of muscle weights, dissected tibia and femur lengths were measured with Vernier callipers.

### Food Consumption

Mice were fed a semi-purified diet based on AIN93G (200 g casein/kg; 70 g soybean oil/kg; metabolizable energy density 3640 kcal/kg; Research Diets). Daily food intakes (averaged over a minimum of 3 d) were measured before and during EE measurements. Food intakes were determined from the change in weight of food cups, corrected for spillage. Food intake during the EE measurements used the automated in-cage feeding assembly of the CLAMS system (Columbus Instruments, described at www.bcm.edu/cnrc/mmru).

### Energy Expenditure

Energy expenditure was measured by indirect calorimetry using a CLAMS Oxymax System (Columbus Instruments). Mice were placed individually into CLAMS cages and after 48 h of adaptation, O_2_ consumption and CO_2_ production were measured for a further 72 h; EE was calculated using the Weir equation [39]. Concurrently, spontaneous physical activity (horizontal and vertical movements) was monitored from the interruption of infra-red beams projected in X and Z axes across the cage. For both EE and activity, only data collected over the last 72 h were analysed. The lowest values of EE over two 90-s periods were averaged and extrapolated over 24 h to provide a measure of resting EE.

### Whole Body Phenylalanine and Tyrosine Fluxes

Whole body protein synthesis and degradation were estimated *in vivo* from measurement of phenylalanine and tyrosine fluxes in a subset of juvenile (n = 12) and adult (n = 10) mice of both strains. Measurements were performed on conscious mice.

#### Infusions and sampling

On the infusion day, feed was removed at 07∶00 and after 3 h a tail vein catheter was inserted as previously described [40]. A primed-continuous intravenous infusion of L-[ring-3,5 ^2^H_2_] tyrosine and L-[ring-^2^H_5_] phenylalanine (prime: 11 and 16 µmol•kg BW^−1^; continuous: 11 and 16 µmol•kgBW^−1^•h^−1^, respectively) was administered to measure the rates of appearance (Ra) of phenylalanine and tyrosine, and the hydroxylation rate of phenylalanine to tyrosine [41]. Blood was sampled after 4 h and the plasma stored at −80°C.

#### Analysis

Plasma phenylalanine and tyrosine isotopic enrichments were determined as their O-phthaldialdehyde (OPA) derivatives by LC MS (TSQ Quantum Ultra System, Thermo Finnigan). Briefly, plasma proteins were precipitated and the supernatants dried under vacuum. Residues were resuspended in ddH_2_O and derivatized with OPA reagents (Agilent Technologies) by the CTC-PAL autosampler (Leap Technologies). Isotopic enrichments were determined by single reaction monitoring the transitions m/z 370 to 220 and 375 to 225 for phenylalanine, and 386 to 118, 388 to 118, and 390 to 118 for tyrosine.

#### Calculations

The rate of appearance of phenylalanine and tyrosine were calculated from the isotopic dilution of the infused tracer at plateau enrichment, as:
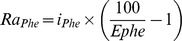
where *Ra_phe_* is the rate of appearance (flux) of phenylalanine (µmol phe•kg^−1^•h^−1^), *i*_phe_ is the infusion rate (µmol•kg^−1^•h^−1^) and *E_phe_* is the enrichment of phenylalanine at plateau (mpe).

The rate of conversion of phenylalanine to tyrosine (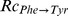) was determined as follows:
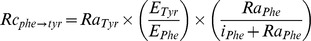

*Ra_Phe_* and *Ra_Tyr_* are the appearance rates of phenylalanine and tyrosine, respectively, determined from the steady state enrichments of the infused tracers; *E_Phe_* and *E_Tyr_* are the respective plasma enrichments of the precursors and products (D_5_ phe and D_4_ tyrosine, respectively).



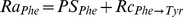



In the fasted state, *Ra_phe_* reflects phenylalanine released from protein degradation which can be used either for protein synthesis (*PS_Phe_*) or hydroxylated to tyrosine (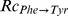). Thus, 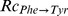 represents irreversible net loss of phenylalanine and in the fasted state measures net phenylalanine balance. To express the phenylalanine flux in terms of protein, we assumed an average phenylalanine content of body proteins of 4% [42], [43].

### Skeletal Muscle Fractional Protein Synthesis Rate

A modification of the procedure described previously was followed [44].

#### *In vivo* tracer study and muscle collection

Briefly, food was removed 7 h prior to the i.v. injection of a bolus flooding dose of L-4-[^3^H]-phenylalanine (American Radiolabeled Chemicals) at 20 mL•kg BW^−1^, 1.5 mmol phenylalanine•kg BW^−1^ and 250 µCi•mouse^−1^. After 15 min mice were euthanized by decapitation and trunk blood collected and acidified. Supernatants were used to estimate the specific radioactivity of blood free phenylalanine. The gastrocnemius and plantaris complex (referred to hereafter as gastrocnemius), quadriceps, soleus, tibialis anterior, diaphragm, and heart were dissected on ice, frozen in liquid N_2_, and stored at −80°C.

#### Muscle analyses

For each muscle, an aliquot of homogenate was retained for measurement of TP. A second aliquot was processed to determine the muscle free phenylalanine precursor pool specific radioactivity, total RNA content, and L-[4-^3^H] phenylalanine incorporated into the muscle TP pool during the labelling period. The MPs were purified from the remainder of the homogenate, and the incorporated L-[4-^3^H] phenylalanine determined. Phenylalanine in acid hydrolysates of the TP and MP fractions, and the muscle and blood supernatants were isolated by anion exchange HPLC (AminoPac1 column, Dionex), post-column derivatized with OPA reagent and detected with an on-line fluorimeter. The radioactivity associated with the phenylalanine peak was quantified by liquid scintillation counting (Packard Tricarb, Perkin Elmer). The phenylalanine concentration was determined by comparing the peak areas of the samples to that of a standard (Pierce Labs.).

#### Calculations

The fractional rate of protein synthesis (FSR), i.e., the percentage of protein mass synthesized in a day, was calculated as:
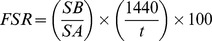
where *S_B_* and *S_A_* are the specific radioactivities of phenylalanine in the protein-bound and precursor pool, respectively; *t* is labelling time in min. Blood and tissue free phenylalanine specific radioactivity values were compared to verify tracer equilibration. Absolute TP synthesis rates were calculated as the product of TP FSR and TP muscle mass; absolute MP synthesis rates were not calculated because the MP purification was not quantitative.

### Protein Concentration

An aliquot of TP muscle homogenate solubilized in 0.3 M NaOH was diluted 1∶40 and protein concentration determined using the micro BCA protein assay (Pierce Labs) with incubation at 60°C for 30 min and bovine serum albumin as standard [45].

### Total RNA

Total RNA was measured quantitatively on all muscle samples to provide an estimate of ribosomal abundance. Total RNA was quantified in the total protein PCA-insoluble precipitate using a modified Schmidt-Thannhauser procedure [46].

### Statistical Analysis

Data were subjected to ANOVA using the general linear model procedure of MINITAB (MINITAB Inc., version 14.2). Data were evaluated for main effects of genotype and age after evaluation for interactions; post hoc testing was performed with Tukey’s method. Values, where appropriate, are expressed as mean±1 SE. To determine if differences among some variables were attributable simply to the difference in body size, ANCOVA was performed using as covariates appropriate measures of body size, e.g. bone or body lengths for body composition outcomes, or FFM and fat mass for energy balance parameters [47], and FFM for phenylalanine flux. When the interaction between the covariate and main effect was significant, data within individual groups were analyzed separately. In these instances values are presented as least square means ± SE mean. For all analyses, individuals performing the work were blinded to the experimental group at the time of the measurement.

## Results

As there were significant interactions between covariates and main effects, data were analysed for each age group separately, and then within genotype across age.

### Body Composition

#### Juvenile mice

Mdx mice were ∼20% lighter and 10% shorter than age-matched controls (Table 1). The weight difference was attributable to proportional reductions in FFM and fat mass; thus, fat as % of body weight was similar for both groups. FFM was highly correlated to body length (r = 0.96), and after accounting for differences in body length, the difference between the FFM of control and mdx mice was not significant (data not shown). Both femur and tibia were significantly shorter in mdx mice and were correlated to total length (P<0.05), but with no genotype x length interaction.

**Table 1:** Measures of body size and composition of juvenile (4- to 5-wk-old) and adult (12- to 14-wk-old) mdx and control mice.

Group	Juvenile		Adult		*P*		
	*mdx*	Control	*mdx*	Control	*A*×*G*	*A*	G
N	8	8	12	10			
***Body Composition***							
Weight (g)	16.5±0.7*	21±0.7*	29.1±0.6	28.6±0.6	<0.001	<0.001	0.03
FFM (g)	14.6±0.4*	18±0.4*	27.5±0.3*	23.7±0.4	<0.001	<0.001	NS
Fat (g)	1.96±0.44	2.3±0.44	2.16±0.36	4.95±0.39*	0.005	0.001	<0.001
(% BW)	11.9±1.2	11±1.2	7.3±1.0*	16.8±1. 1*	<0.001	NS	<0.001
***Lengths***							
Body (mm)	77.7±0.4*	83±0.4*	96.7±0.3	96.3±0.4	<0.001	<0.001	<0.001
Femur (mm)	12.6±0.1*	13±0.1*	16.1±0.1* 1	15.7±0.1^2^	<0.001	<0.001	NS
Tibia (mm)	15.5±0.1*	16±0.1*	18.3±0.1* 1	17.9±0.1^2^	<0.001	<0.001	NS

FFM, fat-free mass. Values are means±SE.

1 n = 8;

2 n = 7,

*significantly different from all groups.

**Figure 2:**
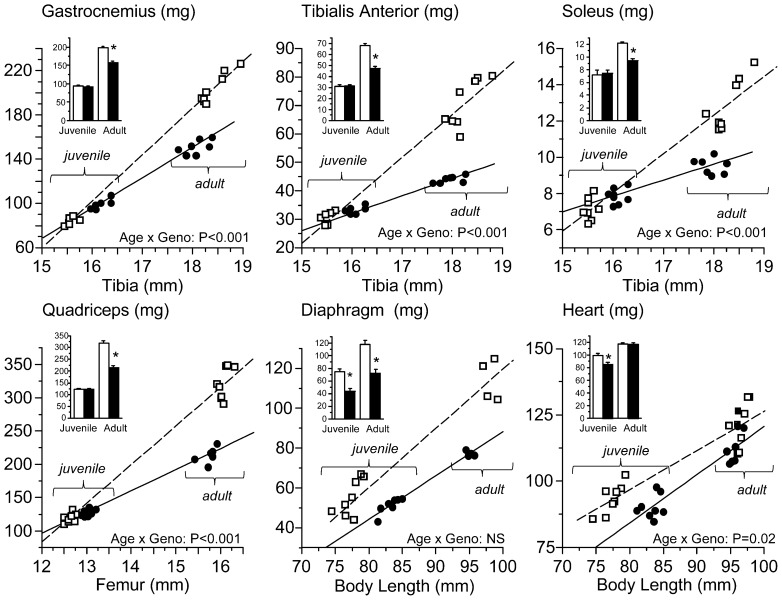
Muscle weights for mdx or control mice relative to bone or body lengths. Individual muscle weights of mdx (□) or control (•) juvenile (4- to 5-wk-old) and adult (12- to 14-wk-old) mice are shown relative to the length of the bone that they subtend; diaphragm and heart are plotted relative to body length. Lines represent the linear regression for values within each genotype (- - -, mdx; ^_____^, control). Insets depict the Least Square Means adjusted for bone or body length±SE at each age for each genotype; *, P<0.05 for mdx vs. control. The data demonstrate that in the juvenile mdx mice the hind limb muscles (gastrocnemius, tibialis anterior, soleus, and quadriceps) are smaller than in controls in proportion to the differences in bone lengths. In the adult mdx mice, the muscles are heavier than in controls regardless of bone length. The diaphragm and heart are significantly heavier in the juvenile mdx mice despite their smaller size. By 12–14 wk of age the diaphragm in mdx mice remains relatively larger than in controls, whereas the heart mass is proportionally similar in both groups.

Absolute weights of all hind limb muscles were significantly lighter in mdx mice and significantly correlated to bone length (Figure 2). The differences in weight were no longer significant when the variation in bone length was accounted for (inserts Figure 2); this was also true with FFM as a covariate. However, TP concentrations were lower in mdx than control muscles (134±4 and 151±3 mg/g muscle, respectively, P<0.01), so that the average TP mass of the gastrocnemius was smaller in mdx mice in absolute terms (Table 2; P<0.001) and also after adjusting for their shorter tibial lengths (P<0.05). Absolute weights of heart and diaphragm were similar for both groups (Figure 2) but after accounting for differences in body length, both muscles were larger in the juvenile mdx mice (inserts to Figure 2). As for hind limb skeletal muscles, the TP concentration of mdx diaphragms was lower than for controls (135±3 and 149±6 mg/g, respectively, P<0.05). Thus, the diaphragm TP mass was similar between genotypes in absolute terms (Table 2), although significantly greater in mdx mice when differences in length were accounted for (genotype x body length, P<0.05). There was no genotype difference in heart TP concentration (156±3 and 158±2 mg/g, respectively, P = 0.4); thus, heart TP mass was similar in juvenile mdx and control mice in absolute terms (Table 2), but significantly greater in the mdx mice when their shorter length (P<0.05) or smaller FFM (P<0.05) are accounted for.

**Table 2:** Fractional protein synthesis rates of muscles from juvenile (4- to 5-wk-old) and adult (12- to 14-wk-old) mdx and control mice.

Group	Juvenile		Adult		*P*		
	*mdx*	Control	*mdx*	Control	*A*×*G*	*A*	*G*
N	8	8	8	7			
***Gastrocnemius***							
TP (mg)	11.1±0.6* † ‡	15.1±0.6† ‡	29.2±0.6*	24.8±0.6	<0.001	<0.001	NS
TP FSR (%/d)	31.1±1.2* † ‡	12.5±1.2†	9.8±1.2*	3.9±1.3	<0.001	<0.001	<0.001
MP FSR (%/d)	24.7±0.9* † ‡	10.4±0.9† ‡	6.7±1.0*	3.2±1	<0.001	<0.001	<0.001
MP/TP FSR	0.8±0.03†	0.83±0.03‡	0.67±0.03*	0.82±0.03	0.075	0.042	0.01
RNA/TP (mg/g)	17.7±0.5* † ‡	10.2±0.5† ‡	7.7±0.6*	5.3±0.6	<0.001	<0.001	<0.001
K_RNA_ (mg/g)	17.6±0.8	12.3±0.8	12.9±0.9	7.2±0.9	NS	<0.001	<0.001
***Diaphragm*** 1							
TP (mg)	7.4±0.4† ‡	7.3±0.4† ‡	15.5±0.5*	11.2±0.5	<0.001	<0.001	<0.001
TP FSR (%/d)	27.3±1.3* † ‡	13.7±1.4†	15.7±1.5*	9.6±1.4	0.011	<0.001	<0.001
MP FSR (%/d)	21.5±0.9* † ‡	11.1±1.0†	10.5±1.1	7.0±1	0.002	<0.001	<0.001
MP/TP FSR	0.79±0.03	0.82±0.03	0.67±0.03	0.74±0.03	NS	0.004	NS
RNA/TP (mg/g)	25.0±1.0	18.9±1.1	18.8±1.7	12.9±1.4	NS	<0.001	<0.001
K_RNA_ (mg/g)	10.9±0.4* ‡	7.2±0.5	9.0±0.7	7.2±0.6	0.1	NS	<0.001
***Heart***							
TP (mg)	14.3±0.5† ‡	14.3±0.5† ‡	19.2±0.5	18.2±0.5	NS	<0.001	NS
TP FSR (%/d)	15.1±0.5* † ‡	12.9±0.5† ‡	8.4±0.5	7.9±0.5	0.011	<0.001	0.011
MP FSR (%/d)	13.6±0.5† ‡	12.9±0.5† ‡	8.1±0.5	8.0±0.5	NS	<0.001	NS
MP/TP FSR	0.9±0.02	0.99±0.02	0.97±0.02	1.01±0.02	NS	NS	0.006
RNA/TP (mg/g)	16.2±0.5	16.4±0.5	12.3±0.5	11.4±0.5	NS	<0.001	NS
K_RNA_ (mg/g)	9.5±0.5* † ‡	7.9±0.5	6.9±0.5	7.0±0.5	0.1	<0.001	NS

TP, total protein; MP, myofibrillar protein; FSR, fractional synthesis rate. Values are means±SE.

1 n = 4 per genotype for 14-wk-old mice. Where interactions are ≤0.1:

*significant genotype (G) effect at the same age (A);

† significant age effect for the same genotype;

‡ significantly different from opposite genotype at older age.

#### Adult mice

There was no difference between BW or length of adult mdx and age-matched controls (Table 1). However, mdx mice were significantly leaner with approximately 15% more FFM and 65% less fat mass. Although FFM was correlated to body length, it was higher in the mdx mice even after normalizing for body length (P<0.001). For all hind limb muscles, both absolute weights and weights normalized for bone length were greater in mdx mice (Figure 2). However, due to the lower TP concentration of mdx gastrocnemius muscle (142±3 and 163±2 mg/g for mdx and control mice, respectively, P<0.001), the difference in TP masses were marginally different after adjusting for tibial length (P<0.06). The TP concentrations for diaphragms were 138±4 and 146±5 mg/g respectively (P<0.05), and the TP mass was higher for mdx mice even after adjusting for body length. Absolute values and height-adjusted values for heart weight, TP concentration (160±2 and 162±2 mg/g respectively), and TP mass were similar in adult mdx and age-matched control mice.

#### Juvenile vs. adult mice

The difference between juvenile and adult mice in body weight and bone length was greater for mdx than control mice. The greater weight gain of mdx mice was due to FFM, as total fat mass was unchanged with age. In contrast, the adult control mice had more than twice as much fat as juvenile mice. The larger muscle mass of adult mdx mice accounted for their higher FFM. At both ages muscle weights overestimated muscle TP content.

### Spontaneous Physical Activity

#### Juvenile mice

**Figure 3:**
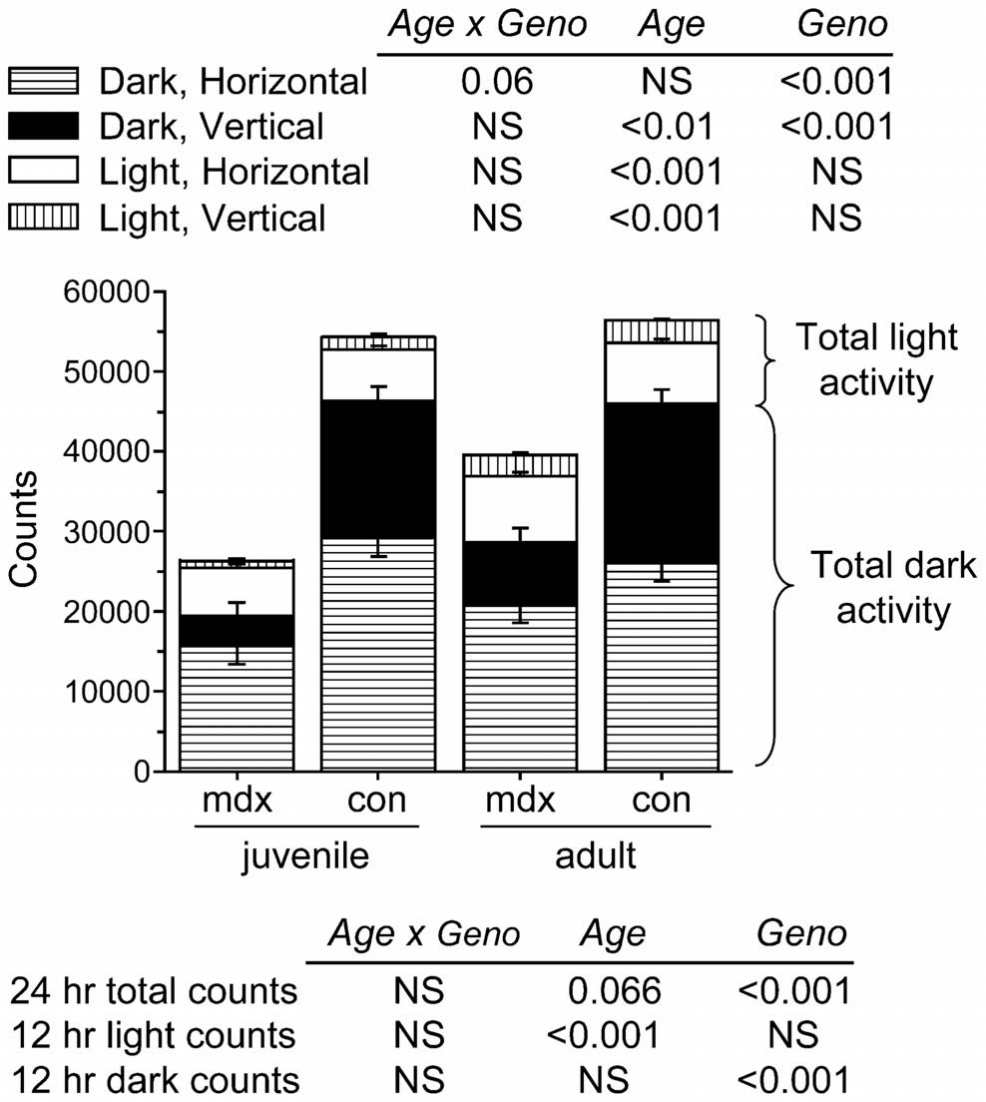
Activity levels over 24 in juvenile and adult mdx and control mice. The height of each column represents the mean value (error bars represent 1 SE; n = 8/group) for the total activity (measured as beam break counts) for each group of mice. Total activity was significantly less in mdx mice at both ages, although the difference decreased with age. The difference in activity levels occurred largely during the dark period when mice are active. At both ages ambulatory (horizontal movement) and rearing (vertical movement) were significantly lower in the mdx than control mice.

Total 24 h activity (Figure 3) was reduced by ∼50% in juvenile mdx mice, and was mainly due to differences in nocturnal behavior when young mdx mice performed only 20% of the vertical and 50% of the horizontal movements of control mice.

#### Adult mice

Total activity was ∼40% lower in adult mdx (Figure 3) compared with control mice and was due to reduced vertical movements at night.

#### Juvenile vs. adult

Total activity increased with age, and the difference was greater for mdx mice. For both genotypes, there was an equivalent increase with age in horizontal and vertical activity in the light phase, and in vertical activity during the dark phase. Nonetheless, total activity remained lower in mdx mice at both ages.

### Food Intake, EE, and Energy Balance

As the same diet was eaten by all mice, differences in energy intakes are entirely explained by differences in daily food intake. Estimates of food intake determined in the CLAMS system closely replicated those measured in home cages.

#### Juvenile mice

Energy intake (kcal/d) was lower in mdx mice compared with controls (P<0.001). This difference was proportional to body size so that when intakes were adjusted for FFM and fat mass, the genotype effect was no longer significant (Table 3). Total daily EE was significantly higher in mdx mice after accounting for differences in FFM and fat. During the dark/active phase, EE was similar for both strains even though mdx mice were significantly less active. During the light/resting phase, when activity levels were similar, EE was significantly higher in young mdx compared with controls. The lowest values for EE invariably occurred during the resting phase, and commonly followed a period of approximately 60 to 90 min when there was minimal food consumption and no activity; therefore, it provides a measure of resting EE. Like total EE, resting EE (adjusted for FFM and fat) was higher for mdx than controls. Daily energy balance, calculated as the difference between energy intake and EE was significantly less positive (∼ 50%) in mdx compared with controls. The average 24 h RQ was similar between genotypes suggesting that both strains were metabolizing the same substrates.

**Table 3:** Parameters of energy balance in juvenile (4- to 5-wk-old) and adult (12- to 14-wk-old) mdx and control mice.

Group	Juvenile				Adult						*P*		
	*mdx*	Control			*mdx*			Control			*A*×*G*	*A*	*G*
N	8	8			9			12					
**Intake**													
24 h (kcal/d)^1^	11.0±0.7	11.3±0.4			11.7±0.6			10.9±0.4			NS	NS	NS
**Expenditure**													
24 h (kcal/d)^1^	11.0±0.5	10.5±0.3			10.4±0.4			10±0.3			NS	NS	0.03
12 h^dark^ (kcal/12 h)^1^	5.9±0.5	5.7±0.2			5.5±0.3			5.6±0.2			NS	NS	NS
12 h^light^ (kcal/12 h)^1^	5.1±0.2	4.8±0.1			4.9±0.2			4.4±0.1			NS	0.04	<0.001
Resting (kcal/d)^1^	9.0±0.4	8.4±0.2			6.5±0.3			6.1±0.2			NS	<0.001	0.01
**Balance**													
24 h (kcal/d)	0.44±0.16*	1.03±0.16			0.74±0.15			0.77±0.13			0.07	NS	0.05
**RQ**													
24 h	0.91±0.02	0.92±0.02 ^‡^			0.95±0.01			0.96±0.01			NS	0.004	NS
12 h^dark^	0.97±0.02	0.97±0.02			0.99±0.02			0.99±0.01			NS	NS	NS
12 h^light^	0.84±0.02	0.87±0.02 ^‡^			0.92±0.02			0.93±0.01			NS	<0.001	NS

EE, energy expenditure; RQ, respiratory quotient.

1 Values are least square means adjusted for differences in FFM and fat mass±SE. Where interactions are ≤0.1:

*significant genotype effect at the same age.

#### Adult mice

Energy intake and total daily EE were both significantly higher in mdx than controls. As in juvenile mice, the energy intakes of mdx and control mice were similar once differences in FFM and fat were accounted for; total daily EE, however, remained higher in mdx mice (Table 3). EE was higher during the dark/active phase than during the day/resting phase, and values were similar for both genotypes despite the higher activity level of control mice. During the light-phase when activity levels were similar for both strains, total and resting EE was greater for mdx mice. Net energy balance was positive in both groups. This result is inconsistent with the cessation of growth, and is likely due to overestimation of food intake caused by underestimation of spillage that was greater for the adult mice. Although there was a significant overall genotype effect on net energy balance, this effect was small in adults compared with juvenile mice. RQ averaged over 24 h was similar between genotypes and higher at night.

#### Juvenile vs. adult

Daily energy intake and EE increased with age in both groups proportional to the increases in metabolic mass. The mass-adjusted 24 h EE was higher in mdx than controls at both ages. Resting EE decreased with age in both groups, independently of changes in metabolic mass. Average RQ increased with age in all mice, and primarily reflected the changes that occurred during the light/resting phase.

### Whole Body Protein Turnover

FFM was included as a covariate in the data analyses to adjust for the effect of the differences in protein pool size across groups.

#### Juvenile mice

**Figure 4:**
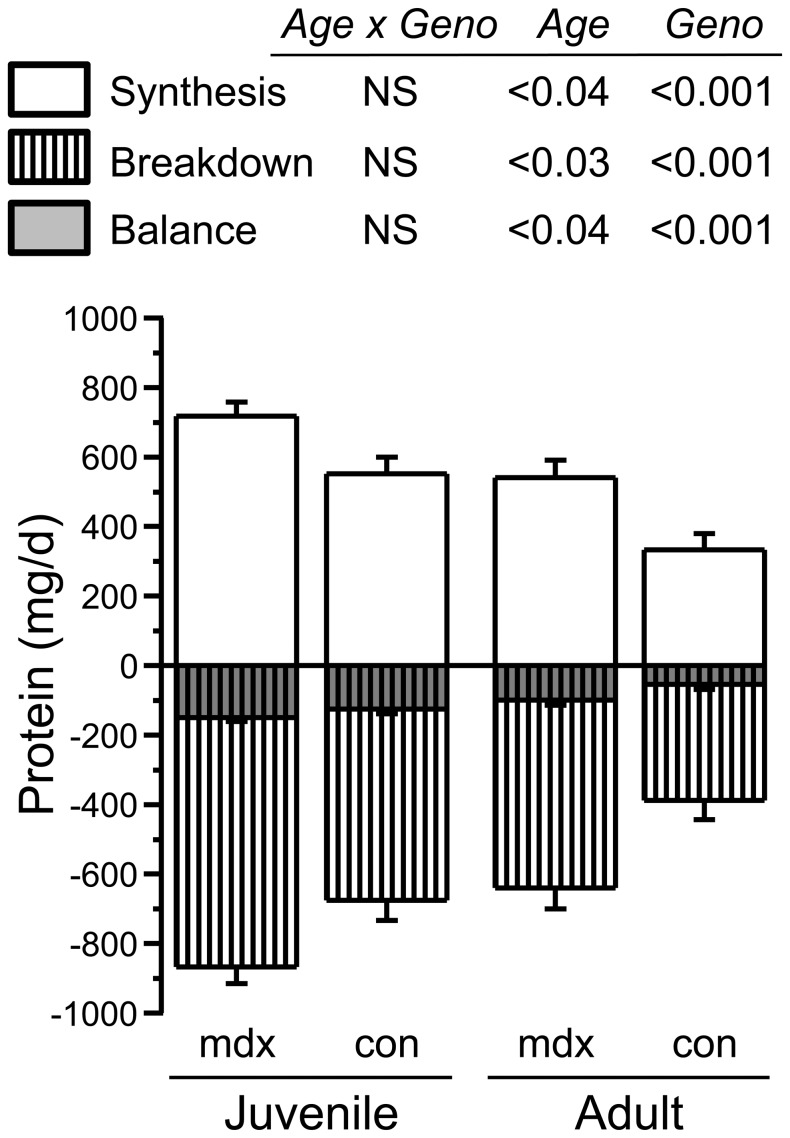
Whole body protein turnover in postabsorptive juvenile and adult mdx and control mice. Whole body rates of protein breakdown, synthesis, and protein balance were estimated from rates of phenylalanine appearance and its conversion to tyrosine. At both ages, rates of synthesis and breakdown were significantly higher in mdx than control mice. Net balance was significantly more negative in mdx mice and positively correlated to phenylalanine flux (r = 0.97). In both mdx and control mice there was a decrease with age in all parameters. Values are least square means (adjusted for FFM) ±SE; n = 5 and 6/group in juvenile and adult mice, respectively.

The rate of phenylalanine appearance, *Ra_phe_*, a measure of whole body protein breakdown, was ∼35% higher in mdx compared with control mice (Figure 4). Because mice were postabsorptive, phenylalanine incorporation into whole body protein (*PS_phe_*) was lower than *Ra_phe_* even though *PS_phe_* was higher in juvenile mdx than control mice, the difference was not sufficient to compensate for their higher protein breakdown rates. Thus, the irreversible loss of phenylalanine, or net balance, was more negative for juvenile mdx than for control mice.

#### Adult mice

In adult mdx mice, Ra_phe_ was almost 60% higher than for controls. As for juveniles, PS_phe_, although also higher in adult mdx mice, did not compensate for the differences in Ra_phe_. Thus, the balance was negative and greater for mdx than control mice.

### Juvenile vs. adult mice

Absolute values for *Ra_phe_* and *PS_phe_* increased with age (data not shown), due to the increased size of the body protein pool with age. Once adjusted for the differences in pool size (as shown in Figure 4), both *Ra_phe_* and *PS_phe_* decreased with age for both genotypes, with no significant interaction. Balance became less negative with age, and was always more negative for mdx than control mice. Phenylalanine hydroxylation to tyrosine represents an irreversible loss, and amounted to 17±2% of phenylalanine flux regardless of genotype or age (r = 0.97 for regression of phenylalanine hydroxylation versus total flux).

### Muscle Protein Synthesis (Table 2)

These measurements were made after completing the food intake assessment, EE, and body composition measurements, by which time the juvenile mice were 32- to 34-d-old, and adults were close to 14-wk-old. Initially, we compared TP and MP FSR for quadriceps and gastrocnemius muscles in a subset of juvenile and adult, mdx and control mice. The values were similar and thus, for the full set of mice, we performed measurements on the gastrocnemius, the diaphragm, and heart.

#### Juvenile mice

FSR of both TP and MP were ∼2-fold higher in gastrocnemius and diaphragm muscles of juvenile mdx compared with control mice; the responses were equivalent for the two muscles (no significant muscle effect). The MPs were synthesized at ∼80% of the TP rate, and the relative contribution of MP to TP FSR was marginally lower in gastrocnemius, but not diaphragm, of mdx mice. For both gastrocnemius and diaphragm, the higher FSRs in mdx mice were attributable to increases in translational capacity (RNA/TP, an indicator of ribosomal abundance) and translational efficiency (K_RNA_). The higher FSR for mdx gastrocnemius was due to a 70% increase in translational capacity and a 40% increase in K_RNA_, whereas for the diaphragm the increases were approximately 30% and 50%, respectively.

The genotype effect on heart protein FSR was less marked than for skeletal muscles. In mdx mice, TP FSR values were ∼20% higher than for controls and this difference was due largely to non-myofibrillar proteins. There was no difference in translational capacity between control and mdx mice; thus, the greater FSR is attributable only to a higher K_RNA_.

#### Adult mice

The gastrocnemius TP FSR values of adult mdx mice were ∼2.5-fold higher than for controls and ∼2-fold higher for the MP fraction. Thus, non-myofibrillar proteins comprised a larger proportion of the proteins synthesized in gastrocnemius muscle of adult mdx compared with controls. The higher FSR for mdx gastrocnemius was the result of a greater translational capacity (40%) and K_RNA_ (80%). Quantitative dissection of the diaphragm was achieved for only 4 mice of each strain. All other parameters evaluated did not differ between this sub-group of mice and the total group. Overall TP and MP FSR values were higher for diaphragm than for gastrocnemius, but the difference in FSRs between genotypes was less than for gastrocnemius (64% for TP; 50% for MP). In contrast to gastrocnemius, the contribution of MP to TP synthesized was similar for mdx and control mice. The increase in translational capacity of the diaphragm was responsible for the greater FSR in mdx mice. There were minor differences in protein synthesis parameters of hearts between adult mdx and controls. Like the gastrocnemius, a smaller proportion of the proteins synthesized were MPs in mdx mice even though, overall, the MPs make a larger contribution to TP synthesis rates in heart compared with skeletal muscles.

#### Juvenile vs. adult

Even though FSR, translational capacity and K_RNA_ were significantly higher in mdx mice at both ages, by and large, they exhibited a developmental decline that was proportionally similar to the change that occurred in control mice. There were some notable differences in mdx muscles: for both gastrocnemius and diaphragm, the age-related decrease in MP FSRs was greater in mdx than controls. For both genotypes, the developmental decline in FSR for gastrocnemius muscle was attributable to decreased translational capacity and K_RNA_. In contrast, for the diaphragm, the differences in FSR were almost entirely due to the decrease in translational capacity with age. The age-related decline in heart TP FSR was greater in mdx mice compared with controls, and reflected a return to normal values from the accelerated values at the younger age. The difference between genotypes in composition of proteins synthesized was maintained. The decrease in heart FSR with age was explained predominantly by decreased translational capacity, and a decrease in K_RNA_ to control values in mdx mice.

## Discussion

The impact of skeletal muscle necrosis and repair on whole body protein and energy metabolism has not been extensively characterized in dystrophic animals or humans. This information is essential in order to assess if the consequences of muscular dystrophy are of a magnitude sufficient to alter the organism’s dietary energy and protein needs for optimal growth and function. Additionally, such data may identify nutritional adjustments that could mitigate disease severity, progression and/or development of secondary complications. A further important consideration is that unless the energy and protein needs of DMD patients are met (which requires that they are accurately defined), the benefit of therapeutic interventions cannot be fully realized. This study identifies significant differences in parameters of EE and protein turnover in mdx mice compared with age-matched controls that had deleterious consequences for growth and body composition. Ultimately, the manifested responses indicate that the diet (optimized to meet the needs of normal animals) did not accommodate the metabolic cost of the disease process in the dystrophic mice especially in juveniles at the onset of dystropathology.

### Growth and Body Composition

FFM in a growing organism is highly correlated to stature. A reduction in linear growth, manifested by the shorter body length of juvenile mdx mice, primarily accounted for their smaller FFM. Similarly, although the limb muscles in mdx mice weighed less than in controls in absolute terms and accounted for their lower FFM, the differences could be explained by their shorter bone lengths. These data indicate that in the juvenile mdx mice overall growth was blunted. A recent survey documents that from as early as 2 years of age, steroid-naıve boys with DMD are shorter on average than the general pediatric male population, suggesting that the metabolic consequences of the dystropathology are similar in both species during early postnatal life [48].

Broadly speaking, reduced growth rates can occur because of inherent biological defects in growth mechanisms; however, the attainment of normal adult size by the mdx mice signifies that their capacity for growth was not inherently defective. Alternatively, growth retardation occurs when nutrient intake is not sufficient to support the growth, maintenance, and activity needs of the organism; the consequences for body composition will depend also on diet composition. The slower growth and reduced FFM accompanied by age-appropriate levels of adiposity in juvenile mdx mice mimics the response observed in young, growing animals fed diets with suboptimal protein:energy ratios where dietary protein intake is limiting relative to energy intake [49]–[51]. Extrapolating these findings to the juvenile mdx mouse, our data suggest that the diet did not contain sufficient protein to support their increased needs for maintenance and growth of FFM.

By 13 to 14 wk of age when growth had ceased, mdx mice were the same weight as controls due to the exclusive gain of FFM. The greater FFM was due to their greater gain in both overall body size and skeletal muscle hypertrophy. With no gain in fat mass, the adult mdx mice were significantly leaner than controls. A lean body composition occurs in normal growing or adult animals fed restricted amount of diets with high protein: energy ratios so that their protein intake is sufficient, but energy intake is limiting [49]–[51]. In these situations, dietary protein intake is sufficient to support growth and/or maintenance needs, as well as being oxidized for energy production to balance expenditure, but with no excess for fat deposition. The body composition of adult mdx mice contrasts with that of many DMD boys who develop obesity [20]. Although differences in energy balance must contribute to differences between mice and humans, diet composition, specifically the relative protein and energy densities of diets, will also influence body composition. The extent to which differences in diet composition in the early stages of the disease contribute to these diverse responses has not been addressed.

Comparison of the TP concentration of mdx and control muscles indicates that at both ages, muscle weight overestimated its metabolically active protein mass. In juvenile mdx the difference is likely due to the greater hydration of dystrophic muscles [52], [53] resulting from local inflammation, myofiber necrosis, and the higher water content of newly formed fibers [54]. In the adult mdx muscle, when there is less active muscle damage and greater muscle fibrosis, TP concentration was likely underestimated by the BCA protein assay we used because it underestimates collagen by approximately 30% when albumin is used as a standard [45]. In mdx muscle the degree of fibrosis varies depending on age and muscle type [31], [55], [56]. Fibrosis in muscles of 4–5-wk-old mdx mice is relatively minor, and would introduce negligible error in the estimates of TP concentration. However, by 3 to 4 mo of age the increased fibrosis might be sufficient to account for the apparently lower TP concentration in adult mdx muscles. The absence of genotype differences in heart TP concentration is consistent with the absence of notable mdx heart pathology at the ages studied [57]. When extrapolated to a whole body level, the differences in the protein content and composition among skeletal muscles can quantitatively impact the interpretation of metabolic data and must be taken into consideration.

### Food Intake and Energy Expenditure

After accounting for their smaller size, total daily EE was higher in juvenile mdx than control mice. Because the juvenile mdx mice did not regulate their intake to match their energy needs, their net energy balance was lower. The imbalance between energy intake and EE during growth explains their reduced weight gain that was already evident by 21 d of age when mdx mice weighed 9.6±0.3 g versus 11.6±0.4 g for controls (P<0.001); in the subsequent 9 d, average daily weight gain was 0.82±0.04 and 0.98±0.02 g/d, respectively (P<0.001). An important difference between juvenile mdx and control mice was their higher resting EE, manifested also in the light/resting-phase EE. In the dark/active-phase, differences in EE were less evident because the greater activity-related EE of control mice compensated for their lower resting EE. In adult mice, due to their larger size and higher tissue metabolic rates, total daily EE also was elevated in mdx compared with control mice. Unlike juvenile mdx mice, energy intake in the adult mdx mice more closely matched daily EE, and the difference in energy balance between genotypes was small. As for juvenile mice, the higher EE in adult mdx mice was reflected in higher values for resting EE during the resting-phase when activity levels were lower. For both genotypes, total EE did not reflect the decrease with age in resting EE most likely because the latter was opposed by the increase in activity with age in both groups.

Variations in resting EE can arise from a number of causes including: the proportion of fat to lean in the body; the masses of tissues and organs with high metabolic rates (liver, gastrointestinal tract, kidney, heart) relative to those with low metabolic rates (normal skeletal muscle, bone, skin) [32], [58]; and changes within a tissue in the rate of cellular processes with a high metabolic cost, such as protein turnover and ion transport. In juvenile mdx mice, the relative amounts of fat and FFM were similar and thus the difference in resting EE persisted after adjusting for body composition. Although heart size was larger in mdx than control mice, the difference was too small to contribute to differences in resting EE [32], [59]. Skeletal muscle weights were proportional to the smaller FFM in juvenile mdx mice, but due to their lower TP concentration, muscle protein masses were reduced. All other factors being equal, a smaller muscle protein mass would augment whole body resting EE, as was observed. Thus, this compositional difference may have contributed in part to the difference in EE between juvenile and control mdx mice. In adult mdx mice, a decrease in resting EE would be expected on the basis of their greater muscle mass; however, their EE was higher even after accounting for their greater FFM. The higher resting EE in both juvenile and adult mdx mice can be anticipated if protein turnover and/or ion transport were elevated. The contribution of the increase in protein turnover to EE is discussed below. With advanced dystropathology, as in older DMD boys, increases in muscle fat content or fibrosis may be sufficient to cancel out the high energy cost processes and to reduce net EE. If not balanced by an equivalent reduction in energy intake this would lead to excess adipose accumulation and obesity.

Increased basal energy requirements can be met by increasing food intake or by repartitioning EE, such as by reducing voluntary activity and growth. Food intake was proportional to body mass for all groups and, therefore, was not responsive to the higher resting EE of the mdx mice. However, the mdx mice reduced their nocturnal activity especially among the juvenile group. These observations are consistent with previous studies which document reduced general activity levels in adult mdx mice [22]. For the juvenile mdx mice, food intake and reduced activity were insufficient to meet energy needs. This imbalance most likely contributed to their blunted growth. By adulthood, when the most severe phase of muscle necrosis has subsided, adaptations in activity and food intake were sufficient to meet energetic demands so that growth was not impaired, but with no excess to promote fat accumulation.

Our EE data contrast with those from a previous study that measured metabolic rate in 4–6-wk-old mdx mice [34]. The difference could be due to differences in ages of mice used and the approaches for normalization of EE data. Our observation of cardiac hypertrophy in the young mdx mice represents a classic functional response to an increased oxygen demand of body tissues and supports our findings of greater EE. Indeed, when we adjusted heart mass for the variation in EE, the genotype effect was no longer significant in juvenile mdx mice, suggesting that larger size may be a functional adaptation. The notion that mdx mice have higher energy needs that cannot be met by a conventional diet is supported by the observation that fasting mdx mice aggravates muscle necrosis [60] whereas feeding mdx mice an energetically dense diet reduces their dystropathology without incurring excessive weight gain [61].

### Muscle and Whole Body Protein Turnover

At both ages, gastrocnemius TP and MP FSR values were ∼2 to 2.5 times higher in mdx than age-matched controls, with proportionally similar decreases with age. These data are consistent with previous reports of high FSRs in muscles of mdx mice [36] and DMD boys [62], [63], although this has not been a consistent finding in humans [64], [65]. The change in gastrocnemius protein FSR with age in mdx mice can be attributed to both the decrease that occurs with muscle maturation in intact regenerated myofibers [66], and also the reduced proportion of myofibers that are hypertrophying and turning over their proteins more rapidly in the early phase of regeneration. The trends were similar for the diaphragm, although the changes in FSR with age were smaller than for the hind limb muscles in both mdx and control mice. In juvenile mice the similar values for MP/TP FSR for mdx and controls indicate that in the mdx muscle, the high FSR of proteins was due to the synthesis of muscle proteins in regenerating myofibers. In adult mdx mice, the difference in TP FSR between mdx and control mice was greater than for MP FSR. The reason for this difference is unclear; it is possible that in the adult, the MP proteins are not impacted to the same extent as sarcoplasmic and membrane proteins and it is the continuous need for synthesis of the latter proteins with inherently higher turnover rates that results in the proportionally higher TP FSR values. It is also possible that at 3–4 mo of age the synthesis of extracellular matrix proteins (measured in the TP sample) accelerates as evidenced by their accumulation in the older muscles.

The effects of muscle dystropathology and age on protein degradation can be inferred from the balance between protein synthesis and accretion rates. Assuming that protein accretion to 8 wk of age is linear [36], we can estimate that at 4 wk of age ∼80% of the gastrocnemius TP synthesized daily was degraded. This represents ∼25% and ∼10% of the gastrocnemius TP mass of juvenile mdx and control mice, respectively. In normal adults protein mass is stable; thus, 100% of the protein synthesized daily is degraded. This represents ∼10% and 4% of muscle TP mass in mdx and control mice, respectively. Thus, ∼60% of the decline in protein degradation rates in the mdx gastrocnemius over this time can be attributed to the decline in muscle dystropathology and the remainder to the normal decrease in protein degradation that occurs with maturation. Using similar assumptions for the diaphragm, relative degradation rates were similar to those for the gastrocnemius muscle in juvenile mice, but were higher in adult mice (∼16 and ∼10% of muscle mass in mdx and controls, respectively). These estimates of degradation are consistent with differences in severity of the dystropathology of the muscles at these ages.

At both ages, the higher FSR of mdx muscle proteins was attributable to a greater translational capacity (dictated by ribosomal abundance, RNA/TP) and translational efficiency (dependent on translation initiation), and these were influenced by muscle type and age. Translational capacity was higher in diaphragm than gastrocnemius mdx muscles at both ages and was largely responsible for the higher FSR of mdx diaphragms. This response may reflect the more oxidative myofiber type composition of the diaphragm, with oxidative myofibers having a higher ribosomal abundance and FSR than glycolytic myofibers [67], [68]. The estimates of translational capacity of the gastrocnemius in both groups of mice were similar to those reported previously [36]. The previously reported values for translational efficiency in mdx mice also were similar to ours, although our values for the translational efficiency for control muscles were lower. The difference most likely reflects the difference between postprandial [36], [69] and post-absorptive FSR (our study) in normal muscles. The ability of mdx muscle to sustain a high protein synthetic efficiency in the post-absorptive state is remarkable because in rapidly growing muscles translation initiation is dependent on activation of the insulin and mTOR signaling pathways that are less active in the post-absorptive state [70]. A possible explanation is that sustained high protein breakdown rates in dystrophic muscles may enable relatively high intracellular amino acids concentrations to be maintained in the post-absorptive state. Thus, unlike control muscles, the amino acid-induced activation of the mTOR pathway that leads to translation initiation may be sustained even in the postprandial state [70]. Similar findings have been documented for other hyper-catabolic conditions [71], [72]. This response is beneficial from the point of view of muscle repair, but it will incur greater EE that will be most evident (relative to controls) when animals are post-absorptive, as indeed we observed in the present study.

The contribution of the higher rate of muscle protein turnover in mdx muscle to whole body EE can be estimated using published data for the contribution of skeletal muscle to whole body O_2_ consumption (30%) and the proportion of resting skeletal muscle O_2_ consumption due to protein synthesis (17%) [32]. In a normal adult animal, skeletal muscle protein synthesis will comprise ∼5% of whole body resting EE. For dystrophic mdx mice, a 2.5-fold increase in the protein synthesis rates of all muscles would increase the contribution of protein synthesis to skeletal muscle oxygen consumption to approximately 33%. After accounting for differences in the muscle protein mass in the body of mdx mice at the different ages (based on gastrocnemius TP masses), the calculated increases in whole body resting EE attributable to higher rates of muscle protein synthesis would be ∼6 and 8% in the juvenile and adult mdx mice, respectively. These values are compatible with the differences we identified in resting EE.

Whole body protein synthesis and breakdown rates followed the same trend as muscle protein, although the differences for whole body measurements between mdx and control mice were proportionally smaller than for muscle. This is to be anticipated if the protein turnover rates in the remaining tissue and organs are unaffected in mdx mice. To our knowledge, only data for the liver have been reported, for which FSR were similar in mdx and controls [36]. At both ages, the mdx mice were in greater negative protein balance, with a trend for values to be more negative in the younger mice. The negative balance represents irreversible loss of phenylalanine, an indispensable amino acid that must be provided by the diet to maintain protein equilibrium. The data demonstrate that phenylalanine released from protein degradation is not recycled with 100% efficiency in the post-absorptive state, and that the proportion catabolized (17%) is constant regardless of age or genotype. Thus, in mdx mice higher flux rates incur greater irreversible loss of phenylalanine. The overall consequence for protein requirements will depend on the metabolic fate of other indispensable amino acids, especially those that are first-limiting in the diet, and on the response of muscle protein turnover to feeding in mdx mice. Nonetheless, the growth and body composition responses observed suggest that the amino acid intake of juvenile mdx mice was insufficient to support age-appropriate rates of growth. Whether this dietary amino acid insufficiency also compromises muscle repair in juvenile mice is a significant issue to address. In adult mdx mice, in which phenylalanine flux was lower, the irreversible losses also were less, and dietary intake was sufficient to support enlargement of muscle mass.

These findings document the metabolic consequences of the muscle dystropathology in mdx mice. While many of the changes can be attributed to differences in cellular composition that result from the age-dependent severity of dystropathology, the data also demonstrate that there are metabolic consequences even when the dystropathology is relatively mild. A dietary intake that does not satisfy the altered metabolic demands of dystrophic muscle could exacerbate the pathogenesis of the condition in addition to blunting growth. Indeed, documentation that linear growth in steroid-naive DMD boys is compromised from a very early age [48], suggests that the metabolic demands of the disease has repercussions for these patients even at a stage when muscle function is only mildly compromised. Our observations on the inter-relationships of growth, protein synthesis rates, energy needs, diet, and activity levels have many implications for understanding the basic pathology of dystrophic muscles, especially with respect to the susceptibility to necrosis of both growing and adult myofibers [24]. These data start to form a rational basis for understanding the protein and energy requirements incurred by muscle disease, as well as the design and impact of various nutritional interventions in mdx mice and DMD boys, with the aim of protecting dystrophic muscle from necrosis and helping to maintain muscle mass at different stages of the disease.
